# Application of BukaGini algorithm for enhanced feature interaction analysis in intrusion detection systems

**DOI:** 10.7717/peerj-cs.2043

**Published:** 2024-04-30

**Authors:** Mohamed Aly Bouke, Azizol Abdullah, Korhan Cengiz, Sedat Akleylek

**Affiliations:** 1 Department of Communication Technology and Network, Universiti Putra Malaysia, Serdang, Selangor, Malaysia; 2 Department of Electrical-Electronics Engineering, Istinye University, Istanbul, Turkey; 3 Department of Computer Engineering, Istinye University, Istanbul, Turkey; 4 Institute of Computer Science, University of Tartu, Tartu, Estonia

**Keywords:** Intrusion detection systems, Feature interaction analysis, BukaGini algorithm, Cybersecurity metrics, Ensemble learning models

## Abstract

This article presents an evaluation of BukaGini, a stability-aware Gini index feature selection algorithm designed to enhance model performance in machine learning applications. Specifically, the study focuses on assessing BukaGini’s effectiveness within the domain of intrusion detection systems (IDS). Recognizing the need for improved feature interaction analysis methodologies in IDS, this research aims to investigate the performance of BukaGini in this context. BukaGini’s performance is evaluated across four diverse datasets commonly used in IDS research: NSLKDD (22,544 samples), WUSTL EHMS (16,318 samples), WSN-DS (374,661 samples), and UNSWNB15 (175,341 samples), amounting to a total of 588,864 data samples. The evaluation encompasses key metrics such as stability score, accuracy, F1-score, recall, precision, and ROC AUC. Results indicate significant advancements in IDS performance, with BukaGini achieving remarkable accuracy rates of up to 99% and stability scores consistently surpassing 99% across all datasets. Additionally, BukaGini demonstrates an average reduction in dimensionality of 25%, selecting 10 features for each dataset using the Gini index. Through rigorous comparative analysis with existing methodologies, BukaGini emerges as a promising solution for feature interaction analysis within cybersecurity applications, particularly in the context of IDS. These findings highlight the potential of BukaGini to contribute to robust model performance and propel intrusion detection capabilities to new heights in real-world scenarios.

## Introduction

In modern cybersecurity frameworks, intrusion detection systems (IDS) are indispensable tools that continuously monitor network activities. They serve the critical role of distinguishing between legitimate operations conducted within the network and unauthorized or malicious intrusions. Despite the remarkable strides made in IDS technology, a formidable challenge persists in accurately pinpointing intrusions amidst the extensive array of authentic network transactions. This challenge is underscored by the crucial aspect of feature selection and the nuanced domain of feature interaction analysis. Indeed, the efficacy of an IDS hinges fundamentally on its adeptness in navigating the intricate interplay of features (Rbah et al., 2022; Eddermoug et al., 2023; Idrissi et al., 2023; Bouke et al., 2023a).

Traditional methodologies employed in IDS have revealed several areas for improvement in handling complex feature interactions, compromising reliability and accuracy in an ever-changing threat landscape. For instance, Gini index-based techniques, initially formulated by Corrado Gini in 1912 for inequality assessments, have been adapted as impurity measures in decision tree algorithms like CART (Classification and Regression Trees). While these approaches offer computational efficiency and reasonable accuracy, they are fraught with limitations such as sensitivity to data noise, suboptimal feature subset selection, and a lack of adaptability to varied scenarios (Gini, 1936; Bouke et al., 2022, 2023a; Mlambo, Chironda & George, 2022; Zhao et al., 2023).

To address these multi-dimensional challenges, this study introduces a novel application of the BukaGini algorithm into IDS, initially developed by Bouke et al. (2023b) for intricate feature interaction analysis within machine learning. Unlike conventional applications, this research explores BukaGini potential to enhance IDS efficacy. The algorithm’s advanced optimization methods and versatile framework are tailored to overcome the limitations of traditional Gini-based methods, promising increased robustness, stability, and interpretability in IDS scenarios.

The primary objective of this research is to demonstrate how the BukaGini algorithm, when applied to IDS, can revolutionize feature interaction analysis, leading to significantly improved intrusion detection accuracy and a reduction in false positives. Moreover, the study addresses the following questions:

1. How does the BukaGini algorithm enhance feature interaction analysis in IDS compared to traditional methods?
2. What impact does the application of BukaGini have on the accuracy and efficiency of IDS?

Through empirical evaluation, this study aims to contribute a transformative approach to IDS, enhancing academic understanding and practical applications in cybersecurity. The overarching goal is to establish BukaGini as an innovative tool for feature interaction analysis, uniquely adapted for IDS challenges.

The remainder of this article is organized as follows: “The Bukagini Algorithm” offers an in-depth exploration of the BukaGini Algorithm, detailing its operational steps and performance metrics. “Literature Review” presents a comprehensive literature review to contextualize the current study within existing research. “Materials and Methods” delineates the research methodology, elaborating on the data preprocessing, model training, and evaluation metrics. “Data Collection” discusses the results compared to traditional feature selection algorithms and explores the implications of these findings. Finally, “Performance Metrics” concludes the article, summarizing the research contributions and suggesting directions for future work.

## The bukagini algorithm

The BukaGini algorithm represents a quantum leap in feature selection by enhancing traditional Gini index-based approaches. It amalgamates three key elements—ensemble learning, feature interaction analysis, and stability analysis—to create a model that surpasses previous performance, generalization, and interpretability limitations. Below, we delve into the intricate facets of the BukaGini algorithm to provide a comprehensive understanding of its workings, merits, and applicability (Bouke et al., 2023b).

The algorithm commences with data preprocessing tasks such as data cleaning, normalization, and other requisite transformations, laying the foundation for robust analysis. Subsequently, the Gini index for each feature is computed, and the features are ranked accordingly. The algorithm employs an ensemble-based approach, leveraging the combined strengths of multiple models to increase robustness and predictive power.

Moving ahead, BukaGini conducts feature interaction analysis to discern and quantify interactions between various features. This crucial step helps identify vital feature interactions that significantly enhance model performance. Stability analysis is the subsequent phase, assessing the robustness and consistency of the chosen features across different data samples. Finally, the algorithm transitions to model training and evaluation, employing a range of performance metrics such as accuracy, precision, recall, and F1 score to validate the selected features.

Moreover, the mathematical framework of the BukaGini is composed of a Dataset *D* consisting of *n* samples and *m* features. Each sample *i* is represented as a vector $\left( {{x_{1i}},\; {x_{2i}}\; ,{\rm \; }...,{\rm \; }{x_{mi}}} \right)$ and is categorized into one of the *c* target classes. The Gini index $Gini\left( j \right)$ For a given feature, *j* is formulated as:

(1) $$Gini\left( j \right)\; = \; 1\; - \; \sum {(P({c_k}|\; {x_j}))^2}$$

**Figure 1:**
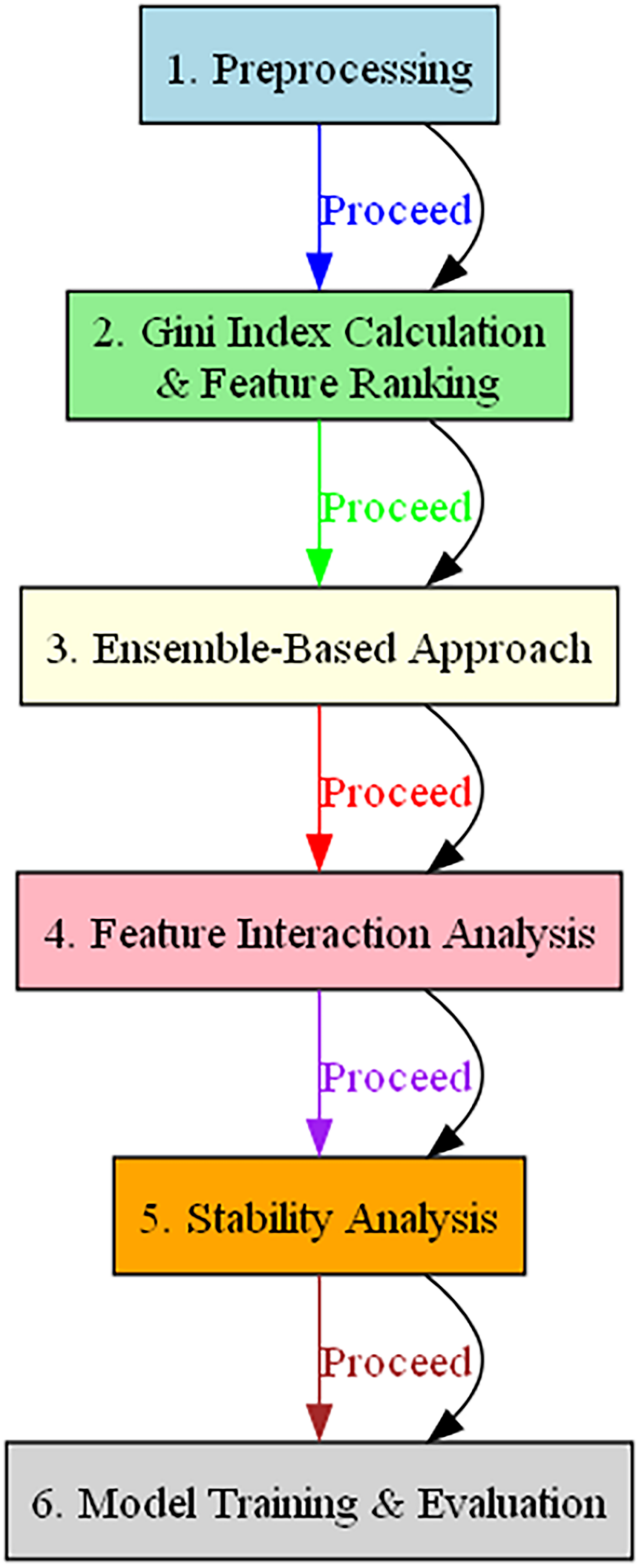
Flowchart illustrating the operational steps of the BukaGini algorithm.

In ensemble learning, the final prediction for each sample is a function *F* aggregating the predictions from *T*-base learners, such as decision trees. For feature interaction analysis, interaction terms like ${I_{j1j2}}$ = ${x_{j1}}$ * ${x_{j2}}$ are introduced into the ensemble model, further enriching its predictive capabilities. On the other hand, the stability analysis is performed through resampling techniques like cross-validation. The average stability score *S* is computed as:

(2) $$S = \displaystyle{1 \over L}*\; \sum C{V_i}$$

where *L* denotes the number of resampled datasets, and $C{V_{i\; }}$ Represents the cross-validation score for the $ith$ resampled dataset. Figure 1 illustrates the BukaGini workflow.

The BukaGini algorithm provides a holistic, enhanced approach to feature selection by melding Gini index-based techniques with ensemble learning, intricate feature interaction analysis, and robust stability assessments. It promises to improve model performance and offer higher interpretability and generalization, positioning it as a formidable tool in diverse application domains, including IDSs.

## Literature review

Intrusion Detection Systems represent a critical frontier in cybersecurity, necessitating relentless innovation in feature selection mechanisms. While existing methodologies have made significant strides, they often need to improve in addressing the complexities of feature interactions. This section aims to provide a comprehensive survey of the existing literature, focusing on the evolution of traditional feature selection methods, the role of neural networks, and the emergence of novel algorithms. Particular attention is devoted to the BukaGini algorithm, a new approach that holds the potential to revolutionize feature selection by capturing intricate feature interactions. Through this review, we lay the foundation for our research, which seeks to empirically validate the efficacy of the BukaGini algorithm in the IDS domain.

Traditional feature selection methodologies serve as the bedrock of IDS research, incorporating statistical models, data clustering techniques, and neural networks. Subba, Biswas & Karmakar (2016) and Can, Le & Ha (2021) have shed light on the capabilities of classical methods. Their research has shown traditional approaches to be generally adequate yet to be limited in terms of scalability and adaptability to new types of intrusion data.

As a vital aspect of IDS, feature selection has garnered much attention. Through optimizing selected features, models can achieve higher levels of accuracy while minimizing computational burden. Seminal works like Di Mauro et al. (2021) and Hassan et al. (2022) have significantly contributed to understanding effective feature selection mechanisms in IDS models. While successful in certain contexts, they have yet to be applicable in scenarios where feature interaction is crucial.

Neural networks offer another dimension to IDS methodologies. Research works from Can, Le & Ha (2021) and Mushtaq, Zameer & Khan (2022) focus on the merits and drawbacks of utilizing neural network algorithms for intrusion detection. These studies indicate that while neural networks are exceptionally good at identifying complex patterns, they are computationally intensive and can sometimes suffer from issues related to overfitting.

As the research landscape evolves, new algorithms are being developed to tackle existing challenges. Preliminary works in this new direction, such as studies from Disha & Waheed (2022) and Kshirsagar & Kumar (2022), reflect a consensus that more nuanced feature selection mechanisms are warranted. These works substantiate the need for innovative algorithms to improve IDS efficiency and accuracy.

In their review article, Bouke et al. (2024), Data Lack, Leakage, and Dimensionality (DLLD) in IDS focus on tackling challenges in IDS related to data scarcity, leakage, and high dimensionality. For data dimensionality reduction, various techniques are discussed. Principal Component Analysis (PCA) is highlighted for reducing large data sets while maintaining accuracy. The article reviews multiple studies that propose diverse methods, such as statistical analysis, and optimization algorithms for effective data management in IDS. These approaches aim to enhance model accuracy and efficiency by addressing the complexity and overfitting issues of high-dimensional data.

Recently, Sarker et al. (2020b) introduced a machine learning method, BehavDT, to establish a user-focused, context-aware predictive framework using a behavioral decision tree. In a related work, they also put forth an Intrusion Detection Tree (called ‘IntruDTree’), an ML-driven intrusion detection system. This system is further enhanced through a meticulously crafted feature ranking and selection strategy (Sarker et al., 2020a). Separately, Al-Omari et al. (2021) have unveiled an astute tree-based model for efficient and effective attack prediction and identificationacks.

Kumar, Almasarani & Majid (2021) delve into the intricacies of integrating 5G technology into smart grid systems, shedding light on its advantages and hurdles. The focal point lies in establishing secure telecommunications networks and fostering information exchange, which is crucial for efficiently managing smart grids. The discourse underscores the imperative for top-notch sensors, computational techniques, and communication infrastructures to enable seamless real-time monitoring and governance. This work serves as a foundational resource for researchers in Saudi Arabia, inviting them to delve into novel smart grid technologies.

Luglio et al. (2023) present the Flexible Web Traffic Generator (FWTG), a specialized tool tailored to simulate the dynamic nature of web-based applications and user engagements. FWTG facilitates the generation of authentic HTTP traffic in real-time, seamlessly injecting it into actual networks, thereby expediting the setup and assessment of network slices within 5G non-public networks (NPN). This tool aids in network design by selecting appropriate backhaul link capacities and defining state-of-the-art services by analyzing traffic patterns and volumes. FWTG showcases remarkable scalability and adaptability in replicating various traffic scenarios, making it an invaluable asset for fine-tuning 5G network setups.

Muheidat, Dajani & Tawalbeh (2022) delve into the strides made by 5G in mobile and wireless technologies while concurrently addressing the imperative for bolstered security measures to mitigate associated risks. The discussion underscores the potential advantages of 5G, including heightened speeds and advanced functionalities. Looking ahead to the era of 6G, the narrative accentuates the pivotal role of quantum computing and networking, particularly concerning fortified security protocols.

Kasongo & Sun (2020) present WFEU-FFDNN, a wireless IDS that amalgamates a wrapper-based feature extraction unit (WFEU) with a feed-forward deep neural network (FFDNN). This novel approach outperforms conventional machine learning algorithms regarding detection accuracy, as evidenced by experiments conducted on UNSW-NB15 and AWID intrusion detection datasets. The results underscore the efficacy of the WFEU-FFDNN methodology, achieving overall accuracies reaching up to 99.77% across binary and multiclass classifications.

Furthermore, the BukaGini algorithm is introduced as an advanced solution for analyzing feature interactions in ML by Bouke et al. (2023b). It surpasses traditional Gini index-based methods in capturing linear and non-linear complex interactions. Quantitatively, the algorithm improves accuracy ranging from 0.32% to 2.50% across four real-world datasets. These datasets span various domains, including student performance, cancer identification, spam email classification, and network intrusion detection. The findings underline BukaGini’s considerable promise in enhancing ML applications across diverse fields. However, its deployment in the IDS domain still needs to be explored. The BukaGini algorithm has the potential for a paradigm shift, mainly due to its ability to consider feature interactions in the selection process.

To this end, IDS represents a pivotal frontier in cybersecurity, demanding continuous innovation in feature selection methodologies. Existing literature showcases a progression from traditional methods to exploring neural networks and developing novel algorithms. Traditional approaches, while adequate, are noted for their limitations in scalability and adaptability. Moreover, studies underscore the significance of effective feature selection mechanisms in enhancing IDS accuracy. Neural networks offer promise in identifying complex patterns but need to be improved by computational intensity and potential overfitting. The emergence of innovative algorithms reflects a consensus on the need for more nuanced feature selection methods to bolster IDS efficiency.

Recent research introduces novel techniques (Table 1), such as BehavDT and WFEU-FFDNN, showcasing advancements in machine learning-driven IDS. Additionally, integrating 5G into smart grid systems and developing specialized tools like the Flexible Web Traffic Generator demonstrate the evolving landscape of cybersecurity infrastructure.

**Table 1:** Algorithm configuration summary.

Reference	Key focus	Methodologies/Algorithms discussed	Main findings/Contributions
Subba, Biswas & Karmakar (2016)	Traditional feature selection methods	Statistical models, data clustering techniques, neural networks	Traditional approaches are adequate but limited in scalability and adaptability
Can, Le & Ha (2021)	Traditional methods, neural networks	Statistical models, neural networks	Neural networks identify complex patterns but are computationally intensive.
Di Mauro et al. (2021)	Feature selection mechanisms	Statistical analysis, feature selection	Effective feature selection mechanisms discussed
Mushtaq, Zameer & Khan (2022)	Neural networks	Neural networks	Neural networks are computationally intensive with potential overfitting issues.
Disha & Waheed (2022)	Novel algorithms	Innovative algorithms	The need for more nuanced feature selection mechanisms highlighted
Kshirsagar & Kumar (2022)	Novel algorithms	Advanced algorithms	Call for innovative algorithms to enhance IDS efficiency and accuracy
Bouke et al. (2024)	Data management in IDS	Principal component analysis (PCA), feature selection, statistical analysis, optimization algorithms	Various techniques discussed for effective data management in IDS
Sarker et al. (2020a, 2020b)	Machine learning methods	BehavDT, intrusion detection tree (IntruDTree)	Introduction of user-focused, context-aware predictive framework and ML-driven IDS
Al-Omari et al. (2021)	Tree-based model for attack prediction	Tree-based model	Astute tree-based model for efficient attack prediction and identification
Kumar, Almasarani & Majid (2021)	Integration of 5G into smart grid systems	Integration of 5G technology, secure telecommunications networks, and information exchange in smart grid systems	Emphasis on secure communication infrastructures for efficient smart grid management
Luglio et al. (2023)	Web Traffic Generator for 5G networks	Flexible Web Traffic Generator (FWTG)	FWTG facilitates simulation of real-time HTTP traffic, aiding in network design and service optimization
Muheidat, Dajani & Tawalbeh (2022)	Security measures in 5G networks	5G advancements, security measures	Discussion on security measures required to mitigate risks associated with 5G
Kasongo & Sun (2020)	Wireless IDS using feature extraction and NN	Wrapper-based feature extraction unit (WFEU), feed-forward deep neural network (FFDNN)	WFEU-FFDNN outperforms conventional algorithms in detection accuracy
Bouke et al. (2023b)	BukaGini algorithm	BukaGini algorithm	The BukaGini algorithm shows promise in capturing feature interactions and improving accuracy across various datasets.

However, despite these advancements, a substantial research gap exists concerning understanding intricate feature interactions in IDS. The BukaGini algorithm, while showing promise in capturing such interactions, still needs to be explored in the IDS domain. This void presents a significant opportunity for further investigation, which this article aims to address. By conducting an extensive study of the BukaGini algorithm within the context of IDS and utilizing common IDS datasets, this article seeks to provide empirical evidence of its efficacy, thereby filling the existing research gap and contributing to the advancement of IDS methodologies.

The novelty of this article lies in its focus on exploring the potential of the BukaGini algorithm in the IDS domain, addressing a significant research gap concerning the understanding of feature interactions. While previous literature has laid the groundwork, more comprehensive studies are still needed. This article aims to fill this void by empirically validating the efficacy of the BukaGini algorithm in IDS, thereby contributing to the advancement of feature selection methodologies in cybersecurity.

## Materials and Methods

**Figure 2:**
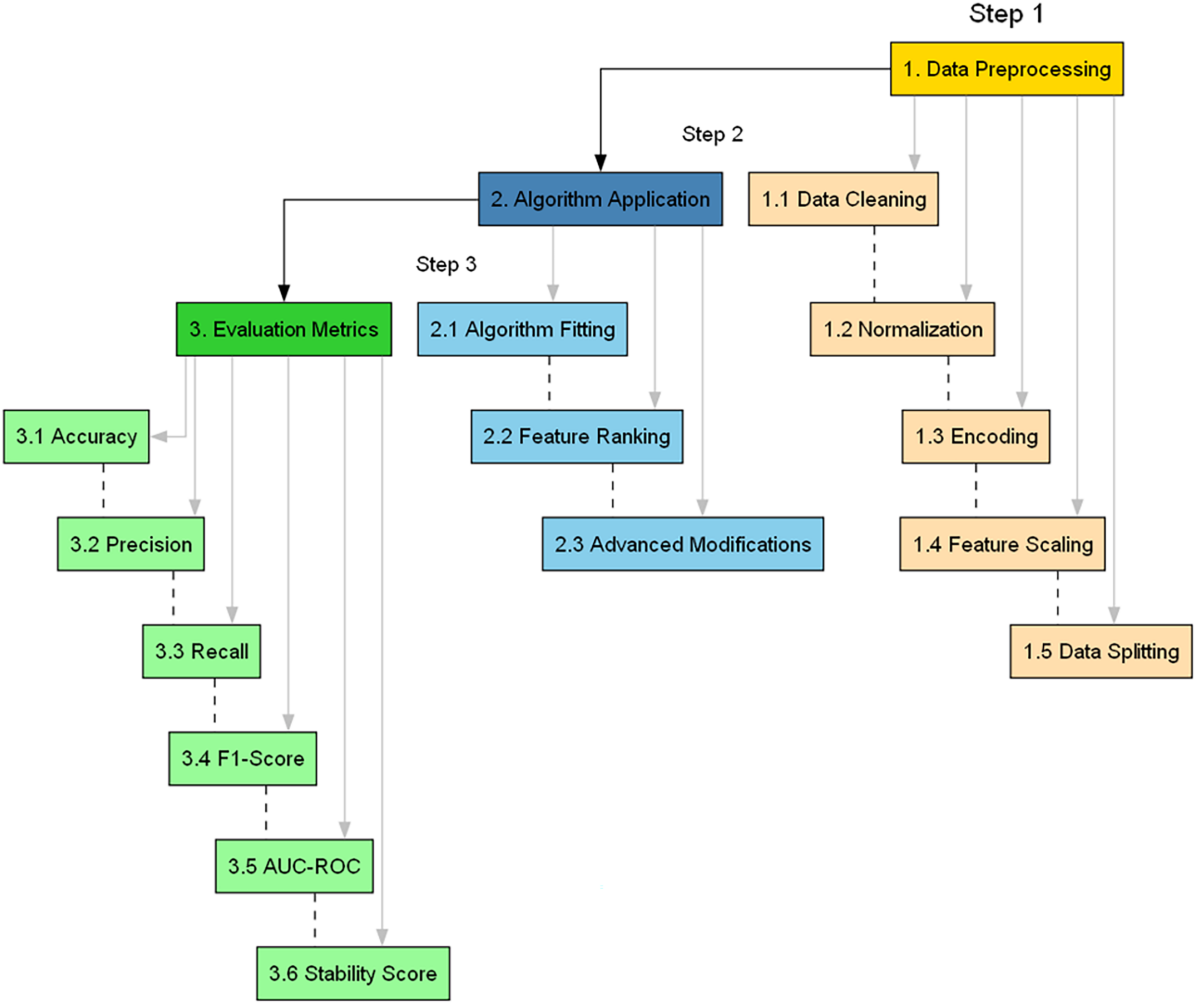
Flowchart of the proposed methodology.

The need for a methodologically rigorous yet adaptable approach must be balanced in Cybersecurity, where the landscape continually evolves. This section provides a comprehensive, detailed, and justified account of the research methods, accentuated by an algorithmic configuration summary. This articulated roadmap is visually summarized in Fig. 2, offering a flowchart of the proposed methodology.

The main steps involved in assessing the applicability of BukaGini in the context of IDS are as follows (Bouke et al., 2023b):

- **Data preprocessing:** The preprocessing journey begins with the crucial step of loading the dataset from a CSV file. This format is chosen for its universality and ease of use, setting a solid foundation for the following stages. The initial loading is more than a mere data import; it represents the first interaction with the dataset, where its structure and characteristics start to unfold. Once the data is loaded, attention shifts to handling missing values. Replacing them with the column mean is a strategic choice, balancing the need to fill gaps while maintaining the original data distribution. This method ensures that the imputation doesn’t skew the dataset’s overall characteristics, essential for maintaining the integrity of subsequent analyses. The next step smoothly transitions into transforming categorical variables. Label encoding is employed, adeptly converting categories into a numerical format. This transformation is pivotal, as many machine learning models inherently require numerical input. The efficiency of label encoding lies in its simplicity and effectiveness, allowing the dataset to integrate with the analytical models in the subsequent stages seamlessly. The final touch in preprocessing is the standardization of features using Standard Scaler. This step is akin to leveling the playing field, where each feature is scaled to have a mean of zero and a standard deviation of one. Standardization is vital; it prevents features with larger numerical ranges from overpowering those with smaller ranges. This ensures that each feature contributes equally to the model’s predictions, safeguarding against potential bias introduced by scale discrepancies. Each step is meticulously designed, providing the dataset is optimally prepped for the BukaGini algorithm’s sophisticated analytical processes. The flow from data loading to standardization is not just a sequence of steps but a thoughtful progression ensuring the data is primed for accurate and effective analysis.
- **Feature importance analysis:** The journey into feature importance analysis begins with RandomForestClassifier, a robust tool chosen for its proficiency in assessing feature significance. This classifier is adept at handling high-dimensional data, making it an ideal choice for this task. Its use in calculating the Gini importance of each feature is a strategic decision, as Gini importance provides a reliable indicator of the value each feature brings to the prediction task. Post Gini importance calculation, the focus shifts to selecting the most significant features—the ‘top-k.’ This selection process is guided by the importance scores, honing in on the features that have the most substantial impact on predictions. The decision to concentrate on the top-k features serves two critical purposes: it reduces the dimensionality of the dataset, making the model less complex and more efficient, and it enhances the interpretability of the model. By narrowing down to the most influential features, the algorithm ensures that the subsequent analysis is manageable and meaningful. This stage of the BukaGini algorithm is crucial, as it lays the groundwork for a focused and effective analysis, ensuring that the most significant features are utilized in the model-building process. The thoughtful application of the RandomForestClassifier in this stage exemplifies a blend of methodological rigor and practical efficiency.
- **Feature interaction analysis:** At this stage, the BukaGini algorithm delves into the realm of feature interactions, an exploration crucial for uncovering the complex relationships within the dataset. By focusing on the top-k features identified in the previous step, the algorithm methodically calculates interaction terms for each feature pair. This intricate process is akin to piecing together a puzzle, where each interaction term adds depth and context to the overall picture. The significance of this step lies in its ability to reveal interdependencies and patterns that might be invisible when considering single features in isolation. These interaction terms serve as new, composite features that encapsulate the joint effect of feature pairs, offering a more nuanced view of the data. Incorporating these interaction terms into the dataset is a strategic move to enrich the feature space. This enrichment is pivotal as it allows the model to capture more intricate patterns, potentially leading to improvements in predictive performance. Adding these terms not only boosts the algorithm’s ability to make accurate predictions but also sheds light on complex feature dynamics that simpler models might overlook. In essence, the feature interaction analysis phase is a testament to the BukaGini algorithm’s sophistication, as it meticulously uncovers and incorporates complex relationships within the data, paving the way for a more robust and insightful predictive model.
- **Stability analysis:** The stability analysis of the BukaGini algorithm is a pivotal stage, where the model’s robustness is rigorously evaluated using k-fold cross-validation. This process involves the RandomForestClassifier, a choice driven by its versatility and reliability in various data scenarios. The essence of k-fold cross-validation is in its methodical division of the dataset into multiple subsets, ensuring that the model is tested and validated across a comprehensive range of data samples. In each fold of the cross-validation process, the algorithm assesses the model’s performance, focusing specifically on the accuracy metric. The mean accuracy score across all these folds is then computed as a critical measure of the model’s stability. This mean score reflects the model’s ability to perform consistently across different data segments, an attribute of paramount importance in the dynamic and unpredictable cybersecurity domain. The choice to use cross-validation is strategic, as it addresses the need for a model that is accurate but also generalizable and reliable in various scenarios. This approach mitigates the risk of overfitting, ensuring that the model’s performance is tailored to a specific subset of data and indicative of its effectiveness across the entire dataset. The stability analysis, therefore, is a testament to the BukaGini algorithm’s commitment to delivering a robust and reliable model, a crucial requirement in the ever-evolving field of cybersecurity.
- **Model training and evaluation:** The culminating stage of the BukaGini algorithm involves preparing the now-enhanced dataset, which includes newly added feature interaction terms. This dataset is meticulously divided into training and testing sets, a standard practice in machine learning to validate the model’s performance on unseen data. The choice of the RandomForestClassifier for training on the training set is strategic, considering its robustness and proven effectiveness in managing complex, high-dimensional datasets. This classifier’s ability to handle intricate data structures makes it particularly suitable for the enriched dataset created by the BukaGini algorithm. Once trained, the model’s performance is evaluated on the testing set using a suite of metrics: accuracy, F1 score, recall, precision, stability score and ROC AUC. Each of these metrics is vital in providing a holistic view of the model’s capabilities. Accuracy measures the overall correctness of predictions, while the F1 score balances precision (the model’s ability to identify positive results correctly) and recall (the model’s ability to find all the relevant cases within a dataset). ROC AUC offers insights into the trade-off between the true and false positive rates, which is crucial for understanding the model’s discriminative ability. This final stage is critical in affirming the BukaGini algorithm’s efficacy, ensuring that the model is accurate but also balanced and reliable across various performance dimensions.

Table 2 offers a summarized view of the BukaGini algorithm configuration to clarify further the choices made at each research stage.

**Table 2:** Summary of implementation environment.

Phase	Technique/Algorithm	Reason for choice
Data preprocessing	Mean value imputation	Maintain overall data distribution
	Label encoding	Efficiently handle categorical variables.
	Standard Scaler	Eliminate feature bias
Feature importance	Gini importance	Identify the most predictive features
Feature interaction	Interaction terms	Capture hidden feature relationships
Stability analysis	k-fold cross-validation	Provide a reliable measure of the model’s stability
Model training	Random forest classifier	Effective for high-dimensional data, handles feature interactions well
Model evaluation	Accuracy, F1, recall, *etc*.	Comprehensive evaluation of the model’s classification abilities

## Data collection

Our research leverages four distinct datasets, each serving a specific purpose and adding nuanced layers of complexity to our evaluation of the BukaGini algorithm in IDS.

The critical evaluation of an intrusion detection algorithm warrants applying a multi-faceted approach. Employing a variety of datasets from disparate cybersecurity contexts provides a robust methodological framework to assess the algorithm’s versatility and effectiveness. In the current study context, we have incorporated four distinct datasets—WUSTL EHMS 2020, NSL-KDD, WSN-DS, and UNSW-NB15—to examine the BukaGini algorithm comprehensively. Each dataset lends itself to a specific set of evaluative criteria, offering a multi-dimensional perspective to the study.

- **WUSTL EHMS 2020 dataset (Unal et al., 2019; Hady et al., 2020):** The WUSTL EHMS 2020 dataset is designed to address the cybersecurity requisites of the Internet of Medical Things (IoMT). Comprising 44 distinct features, the dataset offers a robust platform to probe the BukaGini algorithm’s capabilities in healthcare cybersecurity. The richness of this dataset is particularly pertinent to evaluating the algorithm’s proficiency in handling feature interactions, especially in the nuanced context of IoMT.
- **NSL-KDD dataset (NSL-KDD, 2024; Khraisat et al., 2019):** As an improved iteration of the KDD’99 dataset, the NSL-KDD dataset serves as a conventional benchmark for intrusion detection systems. Utilizing this dataset provides a comparative framework against which the BukaGini algorithm can be evaluated in a well-established academic context.
- **Wireless Sensor Network (WSN-DS) dataset (WSN-DS, 2024; Almomani, Al-Kasasbeh & Al-Akhras, 2016):** Focusing on the intricacies of wireless sensor networks, the WSN-DS dataset offers a fundamentally different set of challenges than traditional network datasets. It is beneficial for evaluating the BukaGini algorithm’s adaptability and performance in distributed systems integral to various Internet of Things (IoT) applications.
- **UNSW-NB15 dataset (Australian Centre for Cyber Security (ACCS); Meftah, Rachidi & Assem, 2019):** Incorporating the UNSW-NB15 dataset allows for assessing the BukaGini algorithm against a broader range of contemporary cyberattacks. This modern dataset offers a realistic panorama of the current cybersecurity landscape, thus making it an essential component for any up-to-date evaluation of intrusion detection algorithms.

Integrating these four datasets into the evaluation framework allows for an extensive, nuanced analysis of the BukaGini algorithm’s performance across various cybersecurity contexts.

## Performance metrics

To ensure a rigorous assessment of the algorithm’s performance, a multi-faceted approach employing various metrics is adopted:

- **Accuracy (Eq. (1)):** Measures the ratio of correct predictions (true positives and negatives) to all predictions. It is an essential metric in classification tasks.

(3) $$Accuracy\; \; = \displaystyle{{\left( {TP + TN} \right)} \over {\left( {TP + TN + FP + FN} \right)}}$$

- **Recall (Eq. (2)):** Calculates the proportion of true positives to all actual positives, also known as sensitivity or the true positive rate.

(4) $$Recall\; \left( {Sensitivity} \right) = \displaystyle{{TP} \over {\left( {TP + FN} \right)}}$$

- **Precision (Eq. (3)):** Computes the ratio of true positives to all labeled positives, also known as the positive predictive value.

(5) $$Precision = TP/\left( {\left( {TP + FP} \right)} \right)$$

- **F-score (Eq. (4)):** A weighted average of precision and recall, considering false negatives and false positives.

(6) $$F \; score = 2*\displaystyle{{\; Precision*Recall} \over {Precision + Recall}}$$

- **ROC AUC (Eq. (5)):** The trade-off between true and false positive rates is evaluated, calculating the area under the receiver operating characteristic curve.

(7) $$TPR\; = \; \displaystyle{{TP} \over {\left( {TP + FN} \right)}}\; \; and\; FPR\; = \; \displaystyle{{FP} \over {\left( {FP + TN} \right)}}\; \;$$

- **Stability Score (Eq. (6)):** this metric assesses the consistency of the model’s performance across different subsets of data, typically calculated in k-fold cross-validation. The Stability Score is the mean of the performance metrics across all folds, indicating average performance and robustness.

(8) $${\rm Stability \; Score} = \displaystyle{1 \over k}\sum\limits_{i = 1}^k {{\rm Metri}{{\rm c}_i}}$$

This Formula (8) ${\rm metri}{{\rm c}_i}$ represents the performance metric (such as accuracy, F1 score, *etc*.) calculated for each fold **i** in the k-fold cross-validation, and **k** is the total number of folds. The formula calculates the mean of these metrics, providing the stability score.

These metrics offer a comprehensive evaluation framework for comparing the proposed algorithm’s efficacy against traditional feature selection methods.

## Implementation environment

The proposed algorithm was implemented in a computational environment well-suited for high-demand tasks. The specifications of the system used for the study include:

- **Operating system:** Windows 10 Pro
- **Hardware:** Intel i7 10th Generation processor, 32 GB of DDR4 RAM, and SSD Storage
- **Programming language:** Python 3.10.9

Key Libraries:

- **Data wrangling:** Pandas
- **Numerical computations:** NumPy
- **ML functions:** Scikit-learn
- **Data visualization:** Matplotlib, Seaborn

For managing this computational environment, Anaconda 3 was the tool of choice. It simplifies package administration and facilitates the easy management of distinct programming environments, thus ensuring the replicability of our experiments.

The development platform for this implementation was Jupyter Notebooks, a web-based application ideal for creating and sharing research documents that include live code, visualizations, and explanatory text.

In summary, the proposed algorithm was evaluated using a comprehensive set of metrics and implemented in a high-performance computing environment, taking full advantage of Python’s extensive ecosystem for scientific computing. Table 3 offers a summarized view of the BukaGini algorithm configuration to clarify further the choices made at each research stage.

**Table 3:** Results of experiments on datasets.

Component	Specification
Operating system	Windows 10 Pro
Hardware	Intel i7 10th Gen, 32 GB DDR4 RAM, SSD Storage
Programming language	Python 3.10.9
Key libraries	Pandas, NumPy, Scikit-learn, Matplotlib, Seaborn
Environment management	Anaconda 3
Development tool	Jupyter Notebooks

## Results and Discussion

In this section, we delve into the outcomes of our evaluation of the BukaGini algorithm’s performance in IDS. Through comprehensive analysis, we assess various metrics to gauge the algorithm’s effectiveness in accurately identifying and mitigating cybersecurity threats. We examine the overall performance results, including stability, accuracy, precision, and other critical evaluation metrics. Subsequently, we explore the algorithm’s proficiency in high dimensionality reduction, elucidating its capability to select relevant features crucial for intrusion detection across diverse datasets. Furthermore, we conduct a comparative evaluation against existing state-of-the-art techniques, providing insights into BukaGini’s superiority and potential enhancements. Finally, we discuss the strengths and weaknesses of the BukaGini algorithm, shedding light on its efficacy and areas for further refinement. This thorough examination aims to elucidate the algorithm’s significance and contributions to advancing intrusion detection capabilities in cybersecurity applications.

### Overall performance results

Table 4 presents the experimental results obtained from the evaluation of the BukaGini algorithm across multiple critical metrics on various datasets. These metrics include stability score, accuracy, F1 score, recall, precision, and ROC AUC, each providing valuable insights into the algorithm’s performance in intrusion detection tasks. By examining these results, we can assess the algorithm’s robustness, effectiveness, and discriminatory power across different datasets, paving the way for a comprehensive discussion of its performance in the subsequent sections.

**Table 4:** Comparative evaluation of BukaGini against state-of-the-art methods.

Metric	NSLKDD	WUSTL EHMS	WSN-DS	UNSWNB15
Stability score	99%	86%	99%	92%
Accuracy	99%	94%	99%	95%
F1 score	99%	94%	99%	95%
Recall	99%	94%	99%	95%
Precision	99%	95%	99%	95%
ROC AUC	100%	96%	99%	99%

**The stability score** is a crucial indicator of an algorithm’s robustness and generalizability across different datasets. In our evaluation, the BukaGini algorithm achieved remarkably high stability scores, ranging from 86% to 99% across the datasets. A Stability Score of 99% on NSLKDD and WSN-DS datasets suggests that BukaGini exhibits exceptional resilience against overfitting, making it highly reliable for practical deployment in real-world IDS scenarios. However, addressing the slightly lower stability scores of 86% on WUSTL EHMS and 92% on UNSWNB15 datasets is imperative. These scores necessitate a deeper examination. The complexity inherent in these datasets, characterized by high imbalance and diverse feature types, could contribute to the observed dip in stability. Despite this, the BukaGini algorithm demonstrates robust performance, showcasing its adaptability to varying dataset characteristics.

**Figure 3:**
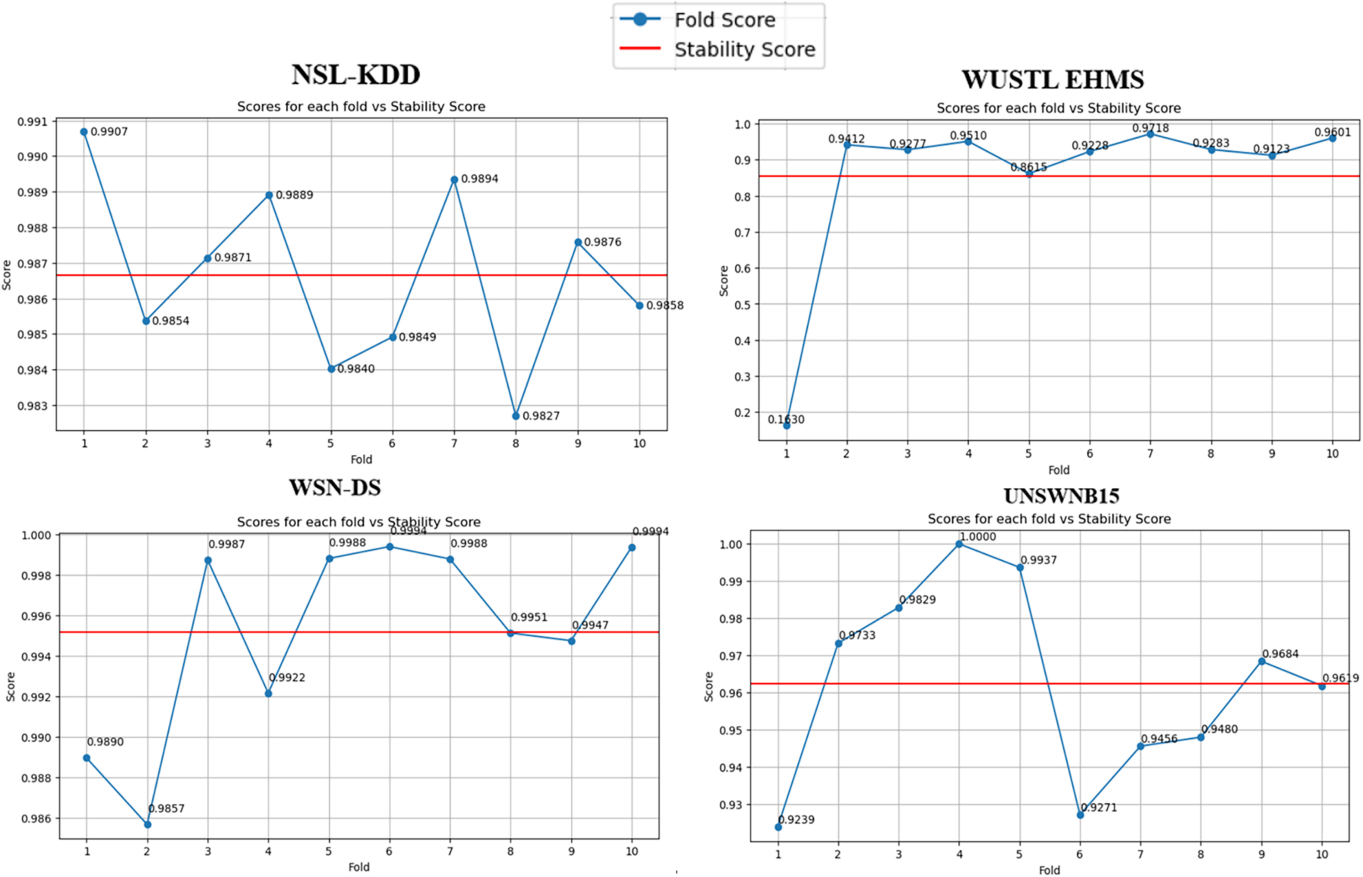
Stability per fold *vs*. scores.

Furthermore, algorithmic stability’s significance across different data folds (10 folds in our case) cannot be overstated (Fig. 3). It serves as a robust litmus test for the algorithm’s generalizability. Our stability analysis revealed that the BukaGini algorithm maintains a high-performance consistency across varying folds of the datasets. This result is another feather in the algorithm’s cap, showcasing its reliability and resilience.

**Accuracy,** as a fundamental metric in classification tasks, holds significant importance in evaluating the performance of an IDS. It quantifies the ability of the system to classify instances as either normal or malicious network activities correctly. The consistent achievement of impressive accuracy rates ranging from 94% to 99% across all datasets by the BukaGini algorithm indicates its robustness and reliability in identifying intrusions accurately. Particularly noteworthy is the commendable 95% accuracy attained on the challenging UNSWNB15 dataset.

The high accuracy achieved by BukaGini underscores its efficacy in distinguishing between normal and malicious network activities, which is essential for reliable intrusion detection in cybersecurity applications. This high accuracy implies that the algorithm can effectively differentiate between benign network traffic and potentially harmful activities, minimizing the risk of false positives and false negatives. In practical terms, a high accuracy rate translates to a reduced likelihood of incorrectly flagging legitimate network traffic as suspicious (false positive) or failing to detect actual intrusions (false negative), thus enhancing the overall effectiveness of the IDS.

The ability of BukaGini to consistently achieve such high accuracy rates across diverse datasets reflects its adaptability and robustness in handling various types of network traffic and intrusion scenarios. This reliability is crucial in real-world cybersecurity environments where the consequences of misclassification can be severe. Therefore, the high accuracy demonstrated by the BukaGini algorithm underscores its suitability for deployment in mission-critical areas where accurate and efficient intrusion detection is paramount.

**The F1 score** is a critical metric in classification tasks, offering a balanced assessment of a model’s performance by considering both precision and recall. Precision measures the proportion of true positive instances among all instances predicted as positive, while recall measures the proportion of true positive instances that the model correctly identified. The F1 score, the harmonic mean of precision and recall, comprehensively evaluates a model’s ability to minimize false positives and false negatives effectively.

In the case of the BukaGini algorithm, its consistently high F1 scores across the datasets, reaching up to 99%, highlight its remarkable performance in balancing precision and recall. Even on the challenging WUSTL EHMS and UNSWNB15 datasets, where the complexity and diversity of features pose significant challenges, the algorithm maintains impressive F1 scores. This indicates its robustness in minimizing classification errors, namely false positives and false negatives.

The ability of BukaGini to achieve high F1 scores reflects its effectiveness in striking a balance between precision and recall, which is crucial for IDS. By minimizing false positives, the algorithm reduces the likelihood of flagging legitimate network activities as suspicious, minimizing unnecessary alerts and reducing the burden on cybersecurity analysts. At the same time, its ability to minimize false negatives ensures that actual intrusions are not overlooked, enhancing the overall reliability of the IDS.

Furthermore, the consistent performance of BukaGini across diverse datasets underscores its adaptability and generalizability in handling various intrusion scenarios. This reliability is essential in real-world cybersecurity applications, where detecting sophisticated and evolving threats demands robust and versatile detection mechanisms. Therefore, the high F1 scores achieved by BukaGini affirm its suitability for deployment in IDS environments, where precision and recall are critical for effective threat detection and mitigation.

**Recall and precision** are pivotal metrics in binary classification tasks, providing valuable insights into a model’s ability to identify positive instances and avoid false positives. In the context of IDS, these metrics hold immense significance, as accurately identifying intrusions while minimizing false alarms is paramount for effective cybersecurity.

BukaGini exhibits robust performance in both recall and precision across all datasets, achieving consistently high rates. Recall, also known as the true positive rate, measures the proportion of actual intrusions the model correctly identifies. On the other hand, precision quantifies the proportion of instances flagged as intrusions by the model that are actual intrusions, thereby reflecting the model’s ability to avoid false positives.

The ability of BukaGini to achieve high rates of both recall and precision underscores its effectiveness in accurately identifying intrusions while minimizing false alarms. In other words, the algorithm demonstrates high sensitivity in detecting intrusions (high recall) while maintaining a low false positive rate (high precision). This capability is particularly crucial in IDS environments, where the consequences of missed intrusions or excessive false alarms can be severe.

By achieving high recall, BukaGini ensures that the majority of actual intrusions are detected, thereby enhancing the overall effectiveness of the IDS in identifying and mitigating cybersecurity threats. At the same time, its high Precision helps minimize false positives, reducing the likelihood of unnecessary alerts and enabling cybersecurity analysts to focus their efforts on genuine threats.

The consistent performance of BukaGini across diverse datasets reaffirms its reliability and suitability for deployment in real-world cybersecurity applications. Its ability to strike a balance between recall and precision ensures effective threat detection while minimizing the risk of false alarms, ultimately enhancing the security posture of organizations and safeguarding against potential cyber threats. Therefore, the robust performance of BukaGini in terms of Recall and Precision solidifies its position as a valuable tool in the arsenal of intrusion detection systems.

The receiver operating characteristic area under the curve (ROC AUC) is a comprehensive metric for evaluating a model’s discriminatory power across various classification thresholds. It provides a holistic measure of the model’s ability to distinguish between different classes, making it a crucial indicator of classification performance. In the context of IDS, where accurately distinguishing between normal and anomalous network behavior is paramount, the ROC AUC holds significant importance.

BukaGini consistently achieves exceedingly high ROC AUC scores across all datasets, ranging from 96% to 100%. This impressive performance underscores the algorithm’s adeptness in effectively distinguishing between normal and anomalous network behavior. The high ROC AUC values indicate that BukaGini exhibits robust discrimination capabilities, allowing it to accurately differentiate between benign network traffic and potential intrusions.

The consistent performance of BukaGini across diverse datasets further highlights its reliability and efficacy in intrusion detection tasks. By achieving high ROC AUC scores, BukaGini demonstrates its ability to effectively separate positive and negative instances across different classification thresholds, thereby consolidating its position as a robust solution for intrusion detection.

Moreover, the high ROC AUC values attained by BukaGini reinforce its suitability for deployment in real-world cybersecurity environments. In such environments, where detecting sophisticated and evolving threats is crucial, accurately discriminating between normal and anomalous network behavior is paramount. BukaGini’s exceptional performance in ROC AUC underscores its effectiveness in addressing this challenge. Further, it solidifies its role as a valuable tool in the arsenal of intrusion detection systems.

**In summary**, the BukaGini algorithm showcases exceptional performance across a spectrum of critical evaluation metrics, thereby highlighting its efficacy in feature interaction analysis within IDS. By consistently achieving high stability, accuracy, precision, and robust ROC AUC scores across diverse datasets, BukaGini emerges as a promising solution for enhancing intrusion detection capabilities in cybersecurity applications.

The algorithm’s ability to maintain high stability underscores its robustness and generalizability across different datasets, indicating its resilience against overfitting and suitability for real-world deployment. This stability ensures the algorithm’s reliability and consistency in detecting intrusions across various network environments.

Furthermore, BukaGini’s impressive accuracy and precision rates reflect its ability to effectively differentiate between normal and malicious network activities while minimizing false positives and negatives. This precision is essential in reducing the occurrence of unnecessary alerts and enabling cybersecurity analysts to focus their efforts on genuine threats, thereby enhancing the overall efficiency of the IDS.

Additionally, the robust ROC AUC scores attained by BukaGini demonstrate its strong discriminatory power in distinguishing between different classes, further solidifying its effectiveness in identifying and mitigating cybersecurity threats. BukaGini contributes significantly to intrusion detection systems’ overall reliability and efficacy by effectively discerning between normal and anomalous network behavior.

Overall, the exceptional performance demonstrated by the BukaGini algorithm across various critical evaluation metrics positions it as a promising solution for enhancing intrusion detection capabilities in cybersecurity applications. Its ability to maintain high stability, accuracy, precision, and robust ROC AUC scores underscores its suitability for real-world deployment. It highlights its potential to improve organizations’ security posture against cyber threats significantly.

### Height dimensionality reduction

The efficacy of the BukaGini algorithm in feature selection is a pivotal aspect influencing its overall performance in intrusion detection. In Table 5, we present a detailed overview of the selected features across various datasets, shedding light on the algorithm’s rationale and choice of attributes deemed most pertinent for effective intrusion detection.

**Table 5:** Selected features overview across datasets.

Dataset	Selected features	Description
UNSWNB15	ct_dst_src_ltm, ct_state_ttl, sttl, rate, sbytes, smean, sload, ct_srv_dst, ct_dst_sport_ltm, dbytes	Number of connections observed between the same (or to the same) destination and source IP addresses, State TTL, Time to live according to the TTL set in the IP packet, rate of packets arriving per second, source bytes, mean size of source packets, load on source, number of connections between the same service and destination port, number of connections for this service and destination port, destination bytes
NSLKDD	dst_bytes, src_bytes, dst_host_diff_srv_rate, dst_host_same_srv_rate, service, dst_host_srv_count, flag, dst_host_rerror_rate, logged_in, duration	Destination bytes, source bytes, different services rate, same services rate, service, destination host srv count, flag, destination host error rate, logged in, duration
WUSTL-EHMS	Flgs, DstJitter, DIntPkt, SIntPkt, Dur, SrcLoad, DstLoad, Packet_num, Load, Sport	Flags, destination jitter, destination packet interval, source packet interval, duration, source load, destination load, packet number, load, sport
WSN-DS	ADV_S, Is_CH, DATA_S, Data_Sent_To_BS, JOIN_S, Expaned Energy, Rank, Dist_To_CH, dist_CH_To_BS, send_code	Advertisement message sent, is a cluster head, data sent, data forwarded to base station, join message sent, expanded energy, rank, distance to cluster head, distance from cluster head to the base station, sending code

In the UNSWNB15 dataset, BukaGini prioritizes features such as **ct_dst_src_ltm** and **ct_state_ttl**, focusing on attributes like the number of connections observed between the same destination and source IP addresses and state TTL. These selections aim to capture patterns indicative of malicious network activities, including unusual connection patterns and abnormal packet rates.

Similarly, in the NSLKDD dataset, the algorithm selects features like **dst_bytes** and **src_bytes**, emphasizing characteristics such as packet sizes and rates of different services. By considering these attributes, BukaGini aims to discern between normal and malicious network behaviors based on distinctive patterns and anomalies in network traffic.

In the WUSTL-EHMS dataset, BukaGini focuses on attributes such as **Flgs** and **DstJitter**, encompassing indicators related to packet flags, jitter, and packet intervals. These selections enable the identification of anomalous network behaviors associated with network activities and communication patterns.

Lastly, in the WSN-DS dataset, features such as **ADV_S** and **Is_CH** are prioritized, reflecting wireless sensor network (WSN) communication attributes like advertisement messages and cluster head designation. By considering these attributes, BukaGini aims to detect anomalies and intrusions in WSN environments by identifying deviations from expected network behaviors.

Overall, the selection of specific features by the BukaGini algorithm underscores its ability to identify and prioritize attributes most relevant for intrusion detection across diverse datasets. By focusing on these key characteristics, BukaGini enhances the accuracy and effectiveness of intrusion detection systems by capturing and analyzing patterns indicative of malicious network activities.

### Work comparison

To substantiate the efficacy and potential of the BukaGini algorithm in the domain of IDS, a detailed comparative evaluation against existing state-of-the-art techniques is crucial. Table 6 presents a comparative analysis, comparing the BukaGini algorithm’s performance metrics with comparable methodologies that employ traditional Gini index-based feature selection techniques on the same datasets.

**Table 6:** Comparative evaluation of BukaGini against state-of-the-art methods.

Work	Accuracy	Precision	Recall	F-score	Dataset	Features
Sarker et al. (2020a)	98.00%	98.00%	97.00%	98.10%	NSLKDD	14/41
Al-Omari et al. (2021)	97.00%	97.00%	97.00%	97.00%	UNSW-NB15	19/41
Bouke et al. (2022)	98.80%	98.80%	98.80%	98.80%	UNSW-NB15	11/41
Ismail et al. (2022)	90%	90%	90%	90%	UNSW-NB15	41/41
Bouke et al. (2023b)	98.00%	98.00%	98.00%	98.00%	UNSW-NB15	13/41
BukaGini	99%	99%	99%	99%	NSLKDD	10/41
BukaGini	95%	95%	95%	95%	UNSW-NB15	10/41

In the study by Sarker et al. (2020a), the algorithm achieved an accuracy, precision, recall, and F-score of 98.00%, 98.00%, 97.00%, and 98.10%, respectively, on the NSLKDD dataset. Similarly, Al-Omari et al. (2021) reported consistent metrics of 97.00% across accuracy, precision, recall, and F-score on the UNSW-NB15 dataset. These results reflect the performance of traditional methods leveraging Gini index-based feature selection techniques.

In comparison, the BukaGini algorithm demonstrated superior performance, achieving a remarkable accuracy, precision, recall, and F-score of 99.00% on the NSLKDD dataset, with a reduced feature set of 10 out of 41 features. This highlights BukaGini’s efficiency in achieving high performance with a reduced feature space, thereby enhancing computational efficiency and reducing complexity without compromising accuracy.

On the UNSW-NB15 dataset, BukaGini achieved an accuracy, precision, recall, and F-score of 95.00%, with a reduced feature set of 10 out of 41 features. Despite using fewer features than previous methodologies, BukaGini maintained competitive performance metrics, demonstrating its effectiveness in feature selection and classification tasks within IDS.

Moreover, the study by Bouke et al. (2022) achieved an accuracy of 98.80% on the UNSW-NB15 dataset, with 11 out of 41 features. BukaGini’s comparable performance on the same dataset with a reduced feature set underscores its efficiency and effectiveness in capturing relevant information for intrusion detection while minimizing computational overhead.

In contrast, Ismail et al. (2022) utilized all available features (41 out of 41) on the UNSW-NB15 dataset, achieving lower accuracy, precision, recall, and F-score metrics of 90.00%. This indicates the potential overfitting associated with using a larger feature set, highlighting the importance of feature selection techniques like those employed by BukaGini.

Furthermore, Bouke et al. (2023a) reported an accuracy of 98.00% on the UNSW-NB15 dataset with 13 out of 41 features. BukaGini’s comparable performance with a reduced feature set reaffirms its efficiency and effectiveness in feature selection and intrusion detection tasks, offering a competitive alternative to traditional methods.

Overall, the comparative evaluation demonstrates the superiority of the BukaGini algorithm in achieving high-performance metrics, such as accuracy, precision, recall, and F-score, while utilizing a reduced feature set. This highlights BukaGini’s potential to streamline feature selection processes, enhance computational efficiency, and improve the effectiveness of intrusion detection systems in cybersecurity applications.

### Strengths and weaknesses

The BukaGini algorithm exhibits several notable strengths in the context of IDS. Firstly, its consistent achievement of exceptional accuracy and precision across all datasets underscores its robustness in accurately discerning between normal and malicious network activities. This capability is pivotal for ensuring the reliability of intrusion detection, as it directly impacts the system’s ability to identify threats while minimizing false alarms effectively.

Moreover, BukaGini demonstrates efficiency in feature selection, as evidenced by its ability to achieve high-performance metrics with a reduced feature set compared to traditional methods. This efficiency enhances computational speed and reduces complexity, offering a streamlined approach to feature analysis without compromising accuracy. By focusing on the most relevant features, BukaGini enhances the overall effectiveness of intrusion detection systems.

The algorithm also showcases robustness against overfitting, as indicated by its consistently high stability scores across different datasets. This resilience suggests that BukaGini maintains a high level of generalizability, making it suitable for deployment in diverse network environments without succumbing to overfitting biases. Such reliability is crucial for ensuring the practical applicability of IDS in real-world scenarios.

Furthermore, BukaGini’s competitive performance, even with a reduced feature set, highlights its effectiveness in capturing relevant information for intrusion detection while minimizing computational overhead. By surpassing or matching the performance of existing methodologies, BukaGini solidifies its position as a promising solution for enhancing intrusion detection capabilities in cybersecurity applications.

Despite its strengths, the BukaGini algorithm also presents several limitations that warrant consideration. Firstly, while it performs exceptionally well on the evaluated datasets, its performance on other datasets or in different network environments still needs to be explored. Further validation across a wider range of datasets would provide a more comprehensive understanding of its effectiveness and generalizability.

Additionally, the efficacy of BukaGini heavily relies on the quality and relevance of the features selected. The algorithm’s performance may be compromised in scenarios where feature engineering is challenging, or features are not well-defined. This dependency underscores the importance of robust feature engineering practices to maximize the algorithm’s effectiveness.

Furthermore, while BukaGini demonstrates superior performance in terms of accuracy and precision, its inner workings may need to be more interpretable. Understanding the rationale behind feature selection and decision-making processes could be challenging, especially in complex network environments. This lack of interpretability may hinder the algorithm’s adoption in settings where transparency and explainability are paramount.

Lastly, the performance of BukaGini may be sensitive to various data characteristics such as class imbalance, noise, or the presence of outliers. Further investigation into its robustness under different data conditions is necessary to identify its limitations and areas for improvement. By addressing these weaknesses, researchers and practitioners can work towards refining and enhancing the efficacy of the BukaGini algorithm for intrusion detection in cybersecurity applications.

## Conclusion

In conclusion, the thorough evaluation of the BukaGini algorithm’s performance in IDS reveals its exceptional efficacy and potential for advancing cybersecurity capabilities. Across various critical evaluation metrics, including stability, accuracy, precision, and feature selection, BukaGini consistently demonstrates robust performance, underscoring its suitability for real-world deployment in diverse network environments.

The algorithm’s remarkable stability, as evidenced by high stability scores across different datasets, reflects its resilience against overfitting and generalizability. Moreover, BukaGini’s ability to achieve high accuracy, precision, and recall rates signifies its proficiency in accurately identifying and mitigating cybersecurity threats while minimizing false alarms.

Notably, BukaGini’s efficiency in feature selection enhances computational speed and reduces complexity without compromising accuracy, thereby streamlining intrusion detection processes. Its competitive performance compared to existing methodologies further solidifies its position as a promising solution for enhancing IDS capabilities.

However, despite its strengths, BukaGini exhibits limitations, including the need for further validation across diverse datasets and environments, dependency on feature quality, interpretability challenges, and sensitivity to data characteristics. Addressing these limitations through continued research and refinement will be crucial for maximizing BukaGini’s effectiveness in real-world cybersecurity applications.

In essence, the BukaGini algorithm represents a significant advancement in intrusion detection technology, offering a potent tool for bolstering cybersecurity defenses. Its robust performance and potential for improvement underscore its importance in the ongoing fight against cyber threats, ultimately contributing to a safer and more secure digital landscape.

## Supplemental Information

10.7717/peerj-cs.2043/supp-1 Supplemental Information 1 Source code of the experiments.

## Additional Information and Declarations

### Data Availability

The following information was supplied regarding data availability: The code is available in the Supplemental File. The datasets are available at: 1. NSL-KDD dataset: https://www.unb.ca/cic/datasets/nsl.html. An improved version of the KDD’99 dataset for network-based intrusion detection systems. It removes redundant records, thus providing a more effective dataset for training and evaluating IDS models. 2. WUSTL EHMS dataset: https://www.cse.wustl.edu/~jain/ehms/index.html. Developed by Washington University, this dataset comes from an Enhanced Healthcare Monitoring System testbed. It is tailored for IoMT security research and includes a variety of cyberattack scenarios. 3. WSN-DS dataset: https://www.kaggle.com/datasets/bassamkasasbeh1/wsnds. A dataset focusing on Wireless Sensor Networks (WSN), available on Kaggle. It’s designed for the development and validation of IDS tailored to WSN environments. 4. UNSW-NB15 dataset: https://research.unsw.edu.au/projects/unsw-nb15-dataset. Compiled by the Cyber Range Lab of the Australian Centre for Cyber Security, this dataset is intended for network intrusion detection, featuring modern attack types in a realistic network traffic scenario.
